# Multicentric, multifocal, and recurrent osteoid osteoma of the hip: first case report

**DOI:** 10.1186/s12891-019-2552-x

**Published:** 2019-04-16

**Authors:** Maria Cristina Cortese, Domenico Albano, Carmelo Messina, Giuseppe Perrucchini, Enrico Gallazzi, Mauro Battista Gallazzi, Primo Andrea Daolio, Luca Maria Sconfienza

**Affiliations:** 1 Istituto di Radiologia, F. Policlinico Gemelli - IRCCS, Università Cattolica del Sacro Cuore, Rome, Italy; 2 Università degli Studi di Palermo, Dipartimento di Biomedicina, Neuroscienze e Diagnostica Avanzata, Palermo, Italy; 3 Dipartimento di Scienze Biomediche per la Salute, Università degli Studi di Milano, Milan, Italy; 4 IRCCS Istituto Ortopedico Galeazzi, Milano, Italy; 5 Ortopedia Oncologica, Fondazione Istituto San Raffaele Giglio, Cefalù, Italy; 6 Università degli Studi di Milano, Scuola di Specializzazione in Ortopedia, Milan, Italy; 7 Struttura Complessa di Radiologia, Azienda Sociosanitaria Territoriale PINI-CTO, Milan, Italy; 8 Struttura Complessa di Ortopedia Oncologica, Azienda Sociosanitaria Territoriale PINI-CTO, Milan, Italy

**Keywords:** Osteoid osteoma, Magnetic resonance imaging, Computed tomography, Radiofrequency ablation, Hip

## Abstract

**Background:**

Osteoid osteoma is a benign bone-forming tumour, which very unfrequently has multifocal or multicentric presentation. We report the first known case of a multicentric, multifocal and recurrent osteoid osteoma treated using radiofrequency ablation.

**Case presentation:**

A 39-year-old man with two-year history of left hip pain was admitted at our Institution. The pain was more intense during the night and partially relieved by salicylates. Pelvis CT demonstrated two lytic lesions (8 and 7 mm, respectively) with surrounding sclerotic reactive bone, both with a central focal area of high attenuation, located in the femoral neck and along the anterior portion of the acetabulum, respectively. Both lesions had clinical and imaging findings consistent with multicentric osteoid osteoma. Thus, the two lesions were biopsied – with pathologic confirmation of osteoid osteoma – and treated using radiofrequency ablation. Hip pain decreased but did not disappear, actually increasing a few months after treatment. CT and MRI were performed showing a smaller lesion (5 mm) with the same imaging features, surrounded by marrow oedema, along the posterior column of the acetabulum. The lesion was considered suspicious for osteoid osteoma, overlooked on previous examinations. Therefore, a diagnosis of multicentric and multifocal osteoid osteoma was established. The new lesion was again treated with radiofrequency ablation with symptom disappearance. However, hip pain relapsed after 18 months, and CT and MRI showed an osteoid osteoma recurrence on the posterior column of the acetabulum, which was biopsied and successfully treated using radiofrequency ablation.

**Conclusions:**

To our knowledge, this is the first reported case of multicentric, multifocal, recurrent osteoid osteoma. Our case report highlights the importance of considering a diagnosis of multifocal osteoid osteoma when dealing with multifocal lytic lesions of the bone and with pain persistence after treatment. It also emphasises the combined role of CT and MRI in this setting.

## Background

Osteoid osteoma (OO) is a benign bone-forming tumour of unknown aetiology that usually affects children and young adults. It accounts for 10–13% of all benign bone tumours and 2.5–3% of all primary bone neoplasms [1]. OO generally develops in long tubular bones, especially in the femur and the tibia. It consists of a mixture of osteoblasts, osteoid, and woven bone, with rich innervation and vascularization. The lesion is generally smaller than one cm, is arranged in a round or oval shape, which is termed as *nidus* [2]. The nidus may have a central region of mineralisation and is surrounded by a variable zone of sclerosis [3]. The most frequent symptom of OO is a strong and well localized pain, which is more intense at night and mitigated by salicylates. Pain could be caused by an intense local inflammatory and vasomotor reaction, with associated sclerosis in the contiguous bone, caused by prostaglandins produced by the tumour [4–6].

Although benign, OO is generally treated with the twofold purpose of removing the pain and to avoid growth disturbance in immature skeletons. Traditionally, treatment relied on surgical curettage or *en-bloc* resection, which are currently considered as invasive procedures. Currently, percutaneous radiofrequency ablation (RFA) is considered the standard of care [6, 7].

OO can be missed on a plain radiograph, especially if small, obscured by reactive bone sclerosis, or located in difficult anatomic locations such as the spine or the pelvis. In this setting, computed tomography (CT) may be helpful in early identification of OO, being considered the imaging modality of choice to detect the nidus and later to guide the RFA procedure [2].

Very unfrequently, OO can be multiple, presenting as multifocal or multicentric lesion [2]. ‘Multifocal’ OO refers to more than one lesion within the same skeletal segment, whereas ‘synchronous multicentric’ OO is related to the simultaneous presence of OO in different bones. Last, a single lesion with more than one nidus is termed as ‘OO with multicentric nidus’ [2].

In this paper, we report a case of a multicentric, multifocal and recurrent OO in a 39-year-old man presenting with 2-year history of left hip pain.

## Case presentation

A 39-year old male presented with a two-year history of spontaneous, intermittent, non-traumatic left hip pain. Symptoms were worsening at night, with no irradiation to the leg, fever, or other associated signs/symptoms. Pain was initially reported as mild, then it worsened and became unresponsive to non-steroidal anti-inflammatory drugs, especially salicylates. At first outpatient examination, diffuse tenderness of the hip was noted, with pain not changing with movement. No palpable swelling or decrease of muscular tone was observed. Hip range of motion was preserved, with negative flexion, abduction and external rotation (FABER) test. No neurological signs were found and Laségue maneuver was negative on both sides.

**Fig. 1:**
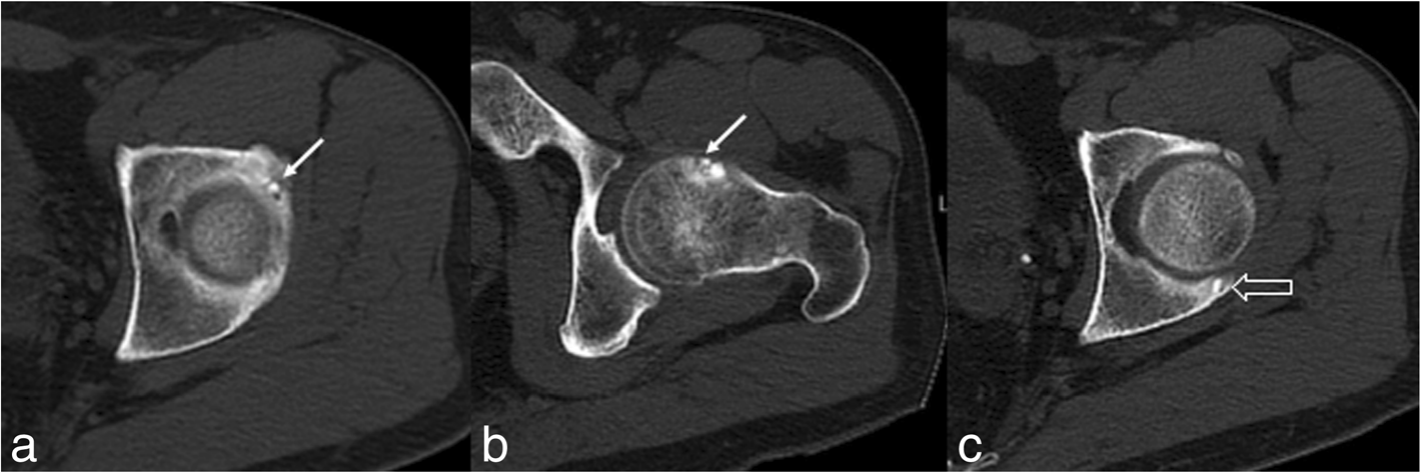
After two-years history of left hip pain, the patient underwent pelvis CT scan revealing the synchronous presence of two intracortical bone lesions consisting of a radiolucent nidus with a central punctiform hyperdensity and sclerosis of the adjacent bone, located in the left anterior acetabulum (**a**, arrow) and femoral neck (**b**, arrow), suspicious for OO. A third smaller lesion of the posterior acetabulum, with the same imaging findings (**c**, void arrow) was initially overlooked

**Fig. 2:**
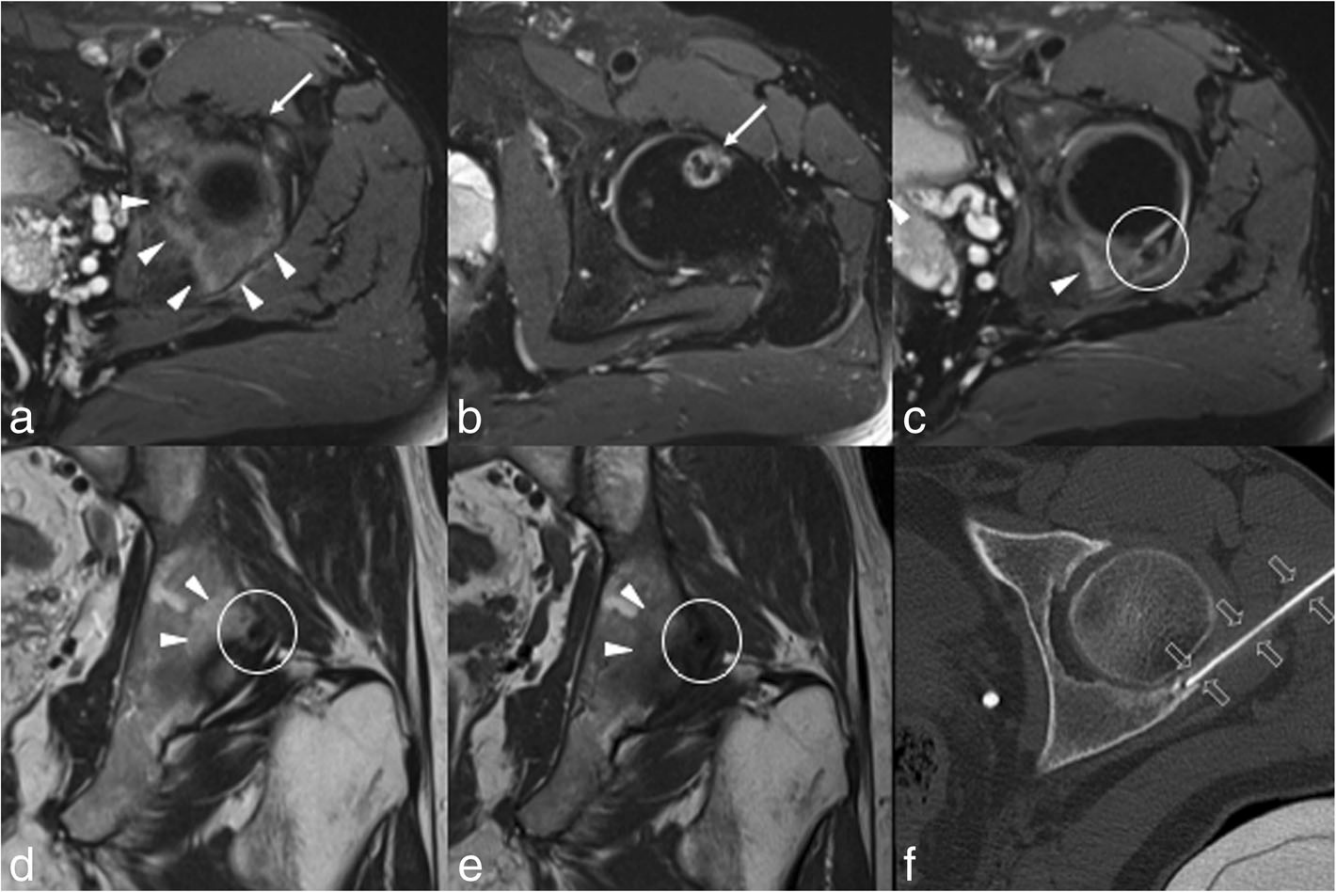
A few months after percutaneous RFA of both lesions and a partial pain relief, the patient complained increased left hip pain and underwent hip MRI (**a**-**e**). Axial fat-saturated proton density-weighted images show the good outcome of the RFA with no marrow oedema on the anterior acetabulum (**a**, arrow) and femoral neck (**b**, arrow). Axial fat-saturated proton density-weighted (**c**), coronal T2-weighted (**d**) and coronal T1-weighted (**e**) MRI images well depict the third bone lesion on the posterior acetabulum (circle) with extensive adjacent bone marrow oedema (arrowheads). Then, CT-guided percutaneous RFA (**f**, open arrows) of the lesion of the posterior acetabulum was performed

**Fig. 3:**
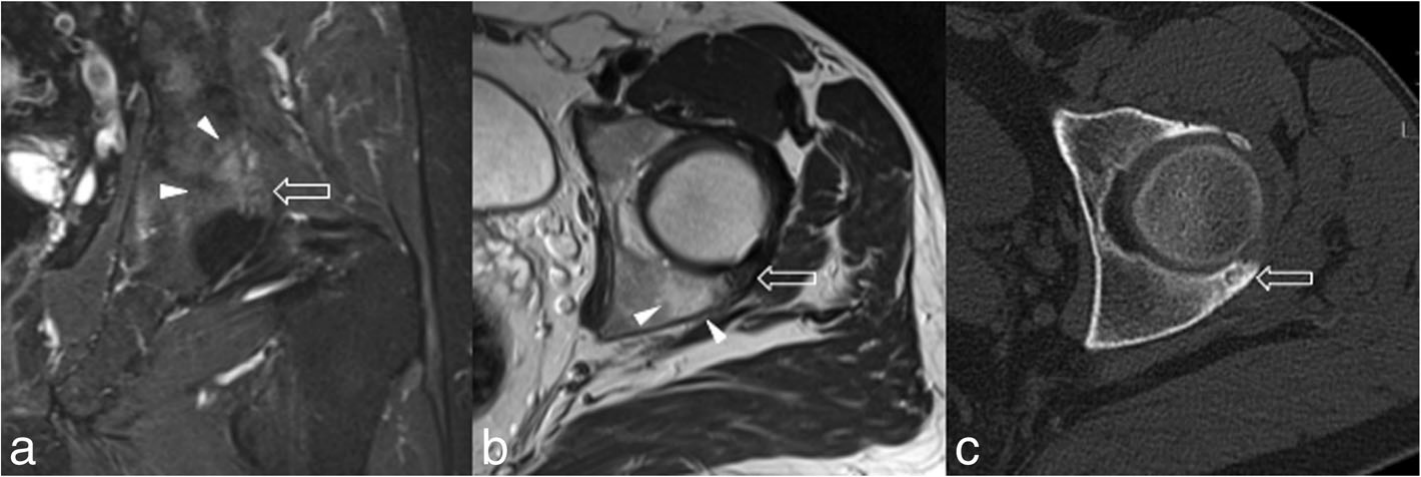
After 18 months, hip pain re-appeared, thus the patient underwent MRI (**a**, **b**) and CT (**c**) showing a recurrence of the previously treated OO of the posterior aspect of the acetabulum (void arrows), with extensive adjacent bone marrow oedema (arrowheads) well demonstrated on coronal fat-saturated proton density-weighted (**a**) and axial T2-weighted (**b**) images

**Fig. 4:**
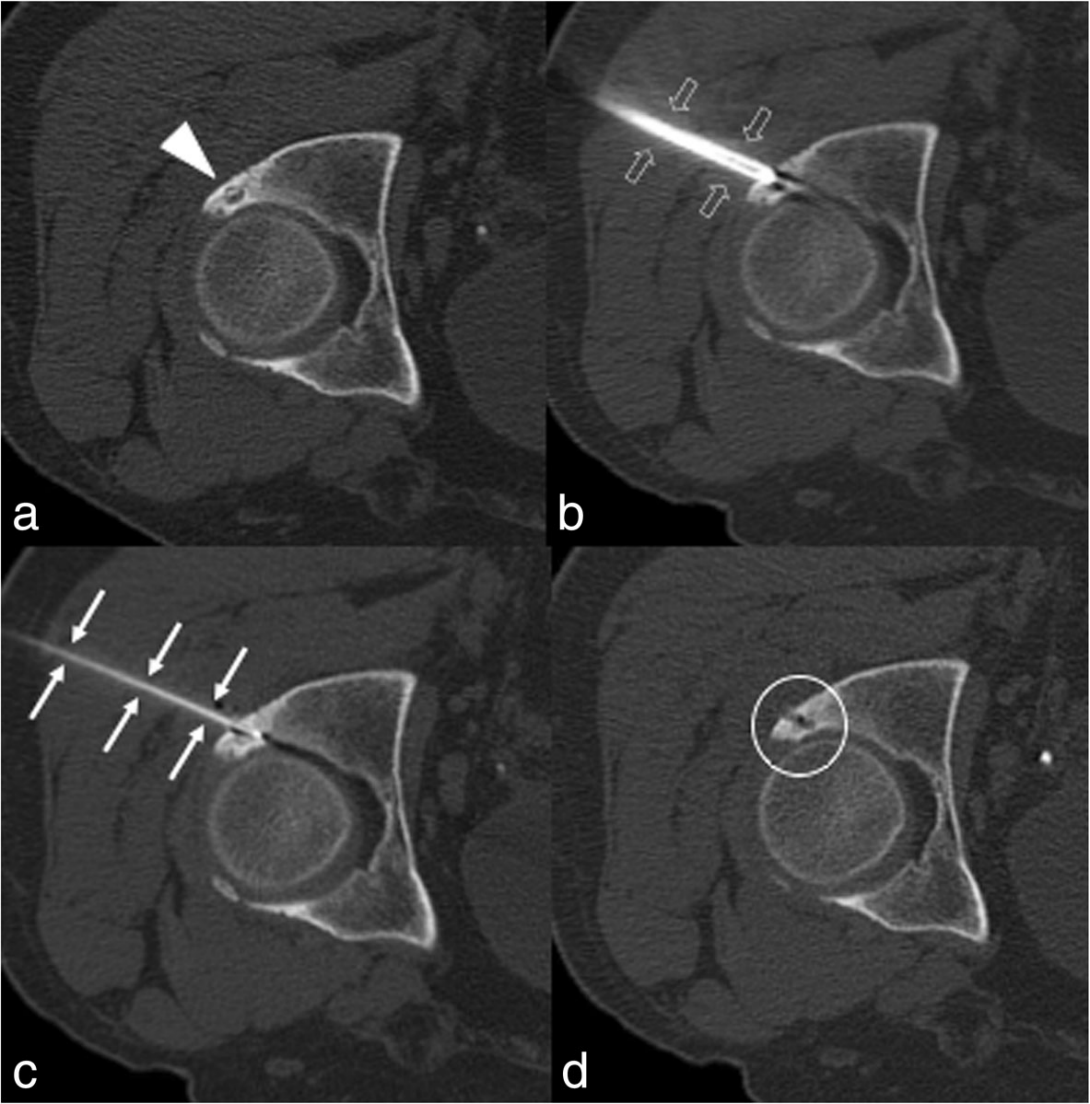
The lesion of the posterior acetabulum (**a**, headarrow) was biopsied (**b**, open arrows) and then treated with RFA (**c**, arrows). Axial CT performed immediately after treatment shows the good results of the ablation (**d**, circle)

The patient underwent pelvis and left hip plain radiography, which was unremarkable. Clinical suspicion of OO was raised and the patient underwent CT examination. It revealed the synchronous presence of an intracortical radiolucent nidus (7 mm) with central hyperdensity and mild sclerosis of the adjacent bone, located in the anterosuperior portion of the left femoral neck. A second lytic lesion (8 mm) with similar features was also seen, surrounded by denser sclerosis, located along the anterior column of the acetabulum. A diagnosis of multicentric OO was made (Fig. 1). Thus, the patient was treated using RFA in a single session. He well tolerated the procedure without complications. Biopsy performed before RFA confirmed the radiological diagnosis of OOs. After treatment, hip pain decreased but did not disappear, actually recurring a few months after treatment. Thus, the patient underwent magnetic resonance imaging (MRI), which showed a smaller lesion (5 mm), along the posterior column of the acetabulum, with imaging features consistent with OO (Fig. 2a-e) and associated to bone marrow oedema. This finding was was overlooked on the previous CT examination (Fig. 1c). Biopsy yielded a diagnosis of OO, which was treated using RFA with disappearance of symptoms (Fig. 2f). Thus, based on the occurrence of two OOs in different part of the same bone and in different bones, a diagnosis of multicentric and multifocal OO was reached. Nevertheless, after 18 months, the patient experienced pain recurrence with the same clinical features as before. Thus, he underwent MRI and CT showing OO recurrence on the posterior column of the acetabulum (Fig. 3). The lesion was biopsied and successfully re-treated with RFA (Fig. 4). Biopsy and pathologic examination yielded again a diagnosis of OO. At present, at 8-month follow-up, the patient is still asymptomatic.

## Discussion

Typically, OO is a solitary, intracortical lesion usually developing in long tubular bones [2], although it may have intramedullary or subperiosteal location, and may arise in any bones [4, 8, 9]. Rarely, OO may have a multifocal presentation and early detection is important to establish an appropriate management [2]. To our knowledge, only four cases of multifocal OO [2] have been reported. Multifocality should be distinguished from multicentricity, a characteristic referring to the development of a primary OO in more than one bone [10]. Only few cases of multicentric OO have been described in literature [3, 4, 6, 10–13].

In our case, the patient was affected by OO which was both multicentric, since it involved the femur and the acetabulum, and multifocal, due to its double localization in the anterior and posterior acetabulum. In addition, recurrence after treatment is another possible and well-known event after OO treatment, easily identified on the basis of the clinical scenarion (pain recurrence) and CT/MRI findings [14]. Frequently, this is the outcome of an incomplete resection procedure rather than true recurrence; however, as in our case, a new RFA treatment may resolve the symptoms [6]. In the presented case, the recurrent lesion in the posterior acetabulum may reasonably represent a consequence of incomplete treatment, either because of technical factors during RFA or the refractory nature of the underlying lesion.

In our case, the third OO located in the posterior acetabulum, which required re-treatment with RFA, was initially overlooked. This is related to the small size and non-specific features of the lesion. Also, this may also be due to a satisfaction of search error due to the identification of the other two lesions, which justified patient’s clinical conditions [15]. Notably, the patient underwent CT only before the first RFA, while MRI was performed just after treatment. Only at that stage, MRI showed the abundant bone marrow oedema in the posterior acetabulum, representing a *“wake-up call”* on MRI scans of patients with OO.

Plain radiography is usually the first line examination, but may result inconclusive especially in cases of intramedullary lesions or tricky locations (e.g. spine, pelvis, hands, and feet). The typical finding is a solid and sclerotic bone reaction, containing a lucent oval-shaped central nidus [14]. CT allows for improved localization of the nidus and it is the imaging modality of choice used to guide RFA procedure [14]. MRI can be used as a problem-solving tool, especially when the lesion arises in uncommon sites, such as juxta-articular or intramedullary [16]. Nevertheless, although sensitive, MRI is not highly specific and is often unable to identify the nidus. Moreover, the adjacent bone marrow oedema seen on MRI may be misinterpreted as signal of aggressive pathology. Furthermore, identification of marrow oedema in the site of RFA is not helpful in the follow-up, as nearly 70% of successfully treated OO present residual oedema at imaging [17]. Thus, a combination of MRI and CT findings may be helpful to rule out doubtful cases.

Surgery of OO has been traditionally used as standard of care. Currently, RFA represent the best treatment option, allowing to treat the nidus by thermal coagulation through a percutaneous applicator, with well documented safety, and success rates higher than 90% [14, 18, 19]. Over the last years, MRI-guided focused ultrasound (MRgFUS) has been proposed as an emerging and non-invasive technique to treat several musculoskeletal disorders, including neoplastic conditions. MRgFUS has shown to be safe and effective for the treatment of OO with promising results in terms of clinical outcomes, recurrence and complications rate [20].

In conclusion, this is the first known case of both multicentric and multifocal OO, also associated with lesion recurrence. Our case report highlights the importance of considering a diagnosis of multifocal OO when dealing with multifocal lytic lesions of the bone and with pain persistence after RFA. It also emphasises the role of combined CT and MRI evaluation.

## Abbreviations

CT: Computed Tomography
MRI: Magnetic Resonance Imaging
NSAIDs: Non-steroidal anti-inflammatory drugs
OO: Osteoid Osteoma
RFA: Radiofrequency Ablation
ROM: Range of motion
